# Solubility, Emulsification and Surface Properties of Maleic Anhydride, Perfluorooctyl and Alkyl Meth-Acrylate Terpolymers

**DOI:** 10.3390/polym10010037

**Published:** 2017-12-30

**Authors:** Marian Szkudlarek, Uwe Beginn, Helmut Keul, Martin Möller

**Affiliations:** 1 DWI Leibniz Institute for Interactive Materials and Institute of Technical and Macromolecular Chemistry, RWTH Aachen University, Forckenbeckstraße 50, D-52056 Aachen, Germany; marian.szkudlarek@ciechgroup.com (M.S.); keul@dwi.rwth-aachen.de (H.K.); 2 Universität Osnabrück, Institut für Chemie, OMC, Barbarastraße 7, D-49076 Osnabrück, Germany

**Keywords:** perfluorinated polymers, self-emulsification, solubility, film formation, contact angle

## Abstract

The solubility of terpolymers containing alkyl, and perfluoroalkyl side chains as well as succinic acid moieties in the main chain, P[R_F_MA_0.2_-*co*-R_H_MA_0.65_-*co*-MAH_0.15_] (R_H_ = C_4_H_9_- or C_12_H_25_-, R_F_- = C_10_H_4_F_19_-) with ca. 20 mol % fluorinated side chains and 10–22 mol % of succinic anhydride rings was tested in a number of solvents varying from water to non polar mineral oils. The polymers are well soluble in fluorinated solvents like Freon-113^®^ and 1,3-bis(trifluoromethyl) benzene, in semi-polar solvents like chloroform, THF or lower esters and also in hydrocarbons with polymers containing dodecyl methacrylate. In self-emulsification experiments, a stable water emulsion of P[F8H2MA_0.2_-*co*-BMA_0.65_-*co*-MAH_0.15_] was obtained. The dispersability and emulsification of these polymers in mixtures of organic solvents and water yielded stable emulsions in the presence of additional surfactant. Thin films coated from organic solutions as well as from emulsions on glass resulted in water and oil-repelling surfaces with contact angles up to 140° against water and 71° against hexadecane. An enhancing effect of annealing was not observed.

## 1. Introduction

**Scheme 1:**
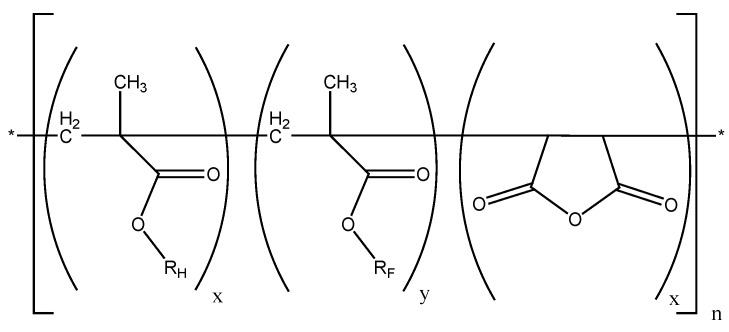
Investigated P[R_H_MA-*co*-R_F_MA-*co*-MAH]_n_ terpolymers (*x* + *y* + z = 1, R_H_ = C_4_H_9_-, C_12_H_25_-, R_F_- = C_10_H_4_F_17_-).

Copolymers with perfluorinated building blocks are desired materials because of their unique properties. The introduction of fluorinated moieties into polymeric materials improves their thermal stability, fastness to outdoor conditions and chemical resistance. One of the most important properties of fluorinated materials is their low surface energy, which allows employing them as water, oil and soil repelling coatings [1,2,3,4,5,6,7,8]. Perfluorinated coatings can be prepared by casting of (i) solutions in organic solvents; (ii) of aqueous polymer emulsions and (iii) of water based solutions/dispersions. The most preferred situation would be the application of a perfluorinated copolymer dissolved or dispersed in pure water, which is the most convenient solution from an economic and environmental point of view. However, the basic idea of forming amphiphilic terpolymer consisting of maleic anhydride and long aliphatic side chain methacrylate has been reported before [9], and a very limited number of publications are found reporting successful self-emulsification of fluorinated polymers in water [1] The creation of water-soluble or water dispersible perfluoroalkyl side chain copolymers is supported by the use of maleic anhydride as a comonomer. Hydrolysis of the succinic anhydride rings in the polymer backbone yields hydrophilic carboxylates [10] that additionally improve the adhesion to any substrate that may interact with carboxylic acid or carboxylate groups [11]. This manuscript presents investigations on the solubility, emulsification and the surface properties of coatings prepared from perfluorinated copolymers of the general structure presented in Scheme 1.

These copolymers were applied on different materials e.g., glass, paper and anodised aluminium. Perfluorinated copolymers that were employed in these investigations were obtained by means of continuous addition terpolymerization of 1*H*,1*H*,2*H*,2*H*-perfluorodecyl methacrylate (F8H2MA), alkyl methacrylates (*n*-butyl methacrylate(BMA) or dodecyl methacrylate (DMA)) and maleic anhydride (MAH).

## 2. Experimental

*Methods:* A Branson Sonifier S-450A ultrasonic processor (Emerson Electric Co., St. Louis, MO, USA) was used to homogenize polymer/water mixtures and prepare emulsions in water. Typical sonication parameters were: 300 W, 90% amplitude.

*Contact angle measurements* were performed by means of the sessile drop method with a Krüss G-2 instrument (Krüss GmbH, Hamburg, Germany), using water and hexadecane as wetting liquids. Samples were prepared by spin-coating of dispersions/emulsions on glass microscope slides (76 × 26 × 1 mm) at 2500 rpm for 20 s. Coatings on anodised aluminium (row offset printing plate provided by Agfa Gevaert NV, Antwerpen, Belgium) were prepared in the same way.

*Polymer samples:* P[F8H2MA_0.2_-*co*-BMA_0.7_-*co*-MAH_0.1_] (C1), P[F8H2MA_0.2_-*co*-BMA_0.65_-*co*-MAH_0.15_] (C2) and P[F8H2MA_0.2_-*co*-DMA_0.58_-*co*-MAH_0.22_] (C3) were synthesized according to the following method described in a detailed way elsewhere [2].

Preparation of the solution A: *n*-butyl methacrylate (0.448 g, 3.15 mmol), 1*H*,1*H*,2*H*,2*H*-perfluorodecyl methacrylate (0.722 g, 1.45 mmol), maleic anhydride (1.33 g, 13.57 mmol) and 2,2′-azo-bis-isobutyronitrile (0.03 g, 0.18 mmol) were dissolved in a mixture of 2-butanone (4.53 g, 3.63 mL) and 1,3-bis (trifluoromethyl) benzene (2.79 g, 3.63 mL). The mixture was placed in a round bottom flask, equipped with a nitrogen inlet, reflux condenser, a magnetic stirring bar and a rubber septum. The reaction mixture was degassed three times by freeze–pump–thaw cycles.

Preparation of the solution B: *n*-butyl methacrylate (11.0 g, 77.46 mmol), 1*H*,1*H*,2*H*,2*H*-perfluorodecyl methacrylate (12.4 g, 23.3 mmol) and maleic anhydride (1.6 g, 16.33 mmol) were dissolved in a mixture of 2-butanone (2.0 g, 2.5 mL) and 3-bis (trifluoromethyl) benzene (2.0 g, 1.45 mL). The solution was degassed three times by freeze–pump–thaw cycles. Preparation of the initiator solution C: 2,2′-azo-bis-isobutyronitrile (0.066 g, 0,4 mmol) was dissolved in mixture of 2-butanon and 1,3-bis (trifluoromethyl)benzene (1.0 g, 1:1 vol:vol). The mixture was degassed by three freeze–pump–thaw cycles.

Continuous addition terpolymerization: The solution A was placed in an oil bath at 65 °C and after 5 min the addition of solutions B and C was started. Solution B was added at a rate of 0.755 mL/h, solution C with a rate of 0.0205 mL/h by means of separate syringe pumps over a period of 33.33 h. After the addition period, the reaction was heated for another 7 h. The reaction mixture was cooled to room temperature and the product was precipitated in methanol (500 mL). 

Characteristics of the copolymers are summarized in Table 1.

*Particle size measurements* were performed using a Malvern Instruments Zetasizer Nano-ZS particle sizer (Malvern Instruments, Malvern, UK). In addition, 2 mL of 2 wt % water solution of the terpolymer C2 was placed in a polystyrene (PS) cuvete without filtration.

*Solubility tests* were performed using the solvents listed in Table 2.

*Emulsification of terpolymers:* The terpolymer (200 mg) was dissolved in an organic solvent (2 mL). Solutions were emulsified in water (20 mL) by means of ultrasonication without or in the presence of sodium dodecyl sulfate (SDS) (50 mg). The final overall concentration of the copolymer was 1 wt %. Table 3 summarises the compositions of the sonicated mixtures.

*Preparation of aqueous solution of C1, C2 and C3:* The copolymer (500 mg) was dissolved in *i*-BuAc (10 g) and added to aqueous ammonia (50 mL, 1%). The mixture was emulsified by means of ultrasonication for 5 min and left for seven days. The spontaneously precipitated copolymer was separated by filtration and transferred to freshly prepared aqueous ammonia (25 mL, 1%). The mixture was homogenized by means of ultrasonication for 10 min followed by stirring at 60 °C for 30 min.

*Pretreatment of glass substrates:* To obtain hydrophilic surfaces of the glass microscope slides (76 × 26 × 1 mm), the substrate was treated for 2 min with a mixture of concentrated sulfuric acid (3 parts) and 30% hydrogen peroxide (1 part). Afterwards, the glass slides were washed with bi-distilled water to remove the acid, and transferred into a glass jar filled with bi-distilled water. 

## 3. Results and Discussion

Three terpolymers, based on 1*H*,1*H*,2*H*,2*H*-perfluorodecylmethacrylate (F8H2MA) *n*-butyl methacrylate (BMA) or dodecyl methacrylate (DMA) and maleic anhydride were investigated: P[F8H2MA_0.2_-*co*-BMA_0.7_-*co*-MAH_0.1_] (C1), P[F8H2MA_0.2_-*co*-BMA_0.65_-*co*-MAH_0.15_] (C2) and P[F8H2MA_0.2_-*co*-DMA_0.58_-*co*-MAH_0.22_] (C3). These copolymers with uniform composition were prepared by means of free radical copolymerization using the continuous flow monomer addition technique [2].

It is expected that the presence of 10–22% of succinic anhydride rings will enhance compatibility with water upon hydrolysis to carboxylate groups. Due to the combination of hydrophilic moieties with hydrophobic alkyl and perfluorinated side chains, these terpolymers should act as surfactants and may exhibit self-emulsifying properties, making them suitable for waterborne applications. 

The coatings prepared from terpolymers C1, C2, and C3 are expected to exhibit hydrophobic and oleophobic properties due to the presence of 20% of perfluorinated side chains. The perfluorinated side chains should orient towards the air interphase of the polymeric layer and thus strongly influencing the surface properties. This orientation should be supported by the presence of alkyl side chains due to the so-called stratification effect [12,13]. This term denotes a spontaneous formation of separate layers of inherently incompatible components (e.g., fluorinated and non-fluorinated) in polymer films whenever the molecular mobility is promoted by a temperature exceeding the glass transition temperature or in the presence of a solvent. The effect of the length-ratio of alkyl- to perfluoroalkyl side chains on the stratification process should become visible by comparison of C1 and C2 (*n*-butyl) with C3 (*n*-dodecyl side chains). 

*The Solubility of C1–C3* in different solvents was tested prior to any further experiments (Table 4). Polymer samples were added in small portions to 2 mL of solvent while stirring. In cases where the copolymers were highly soluble, the experiment was ended at a concentration of ~20 wt %, and the upper limit of solubility was not further checked except from the case of C1/MEK where the saturated solution contained 68 wt % of C1. In case of experiments where the upper solubility limit was exceeded, the insoluble material was separated by filtration, the filtrate was dried, and the mass of the residue was used to calculate the solubility. 

The highest solubilities (>20 wt %) of the investigated polymers was observed in chloroform (S1), THF (S2), esters (S3, S5), MEK (S4) as well as perfluorinated solvents like hexafluoroxylene (HFX) (S6) or Freon-113^®^ (S7).

In case of insolubility of the polymer in the used solvent, the residual mass is given according to the accuracy of the balance. The butyl methacrylate containing copolymers swell without dissolution in 2-octyl-1-dodecanol (S20) and dipropylene glycol monomethyl ether (S21), while, in non-polar alkanes (S8–S13), the polymers C1 and C2 are practically insoluble (<0.01 wt %). None of the copolymers is soluble in mineral (S14–S16) or silicon (S17) oils as well as in water (S19) and aqueous ammonia (S18).

The solubility properties of the dodecyl methacrylate containing polymer C3 are quite similar to the butyl methacrylate copolymers C1 and C2 with the important difference that C3 is well soluble in toluene (S8), mesitylene (S9), cyclohexane (S13) and n-alkanes (S10–S12). This can be explained by the relatively high content of long alkyl chains, which make the copolymer of dodecyl methacrylate more compatible with hydrocarbons. 

**Scheme 2:**
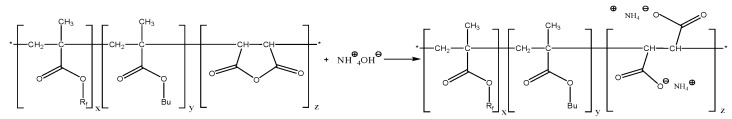
Hydrolysis of the succinic anhydride ring with ammonia solution yields ammonium carboxylate groups.

An aqueous solution of the terpolymer, which contains perfluorinated and alkyl chains, may be obtained by hydrolysis of the succinic anhydride repeating units. The succinic anhydride ring was expected to hydrolyze in water (S19) or aqueous ammonia (S18) to give hydrophilic carboxylate moieties (Scheme 2).

None of the three investigated terpolymers spontaneously dissolved in water or in aqueous ammonia. Most probably, the MAH content is too low; alternatively, the accessibility of the anhydride moiety might not be sufficiently high to allow for their hydrolysis.

In summary, one can state that a moderate content of fluorinated comonomer units results in good solubility of the terpolymer in a wide range of semi-polar solvents, mainly non-halogenated solvents. It has also been demonstrated that the choice of the non-fluorinated comonomer enables enhanced solubility in a specific type of solvent e.g., long alkyl chains enhance the solubility in hydrocarbons. This leaves a certain freedom for designing polymers with better solubility in a solvent of choice.

*Polymer coatings by solution casting*: In a first series of coating experiments, the C1 and C2 terpolymers were coated from 1 wt % organic solution on glass substrates. The advancing contact angles of water and hexadecane as wetting liquids were measured at 20 °C by means of the sessile drop technique with the dried films prior and subsequent to an annealing step. The annealing was performed on dry coating in an oven at 120 °C for 12 h. The contact angles of the coatings obtained from chloroform solutions, as summarized in Table 5, did not change upon annealing (cf. S1-C1, S1-C1 * and S1-C2, S1-C2 *). 

Hence, during the film formation process, the mobility of the side groups was sufficient to ensure proper orientation of the fluorinated side chains towards the polymer/air interface even at ambient temperature. Since in any case the glass transition temperature *T*_g_ of the pure polymers (29–58 °C, see Table 1) were higher than the used coating temperature, the presence of a solvent induced “plasticizer-effect” was concluded.

**Figure 1:**
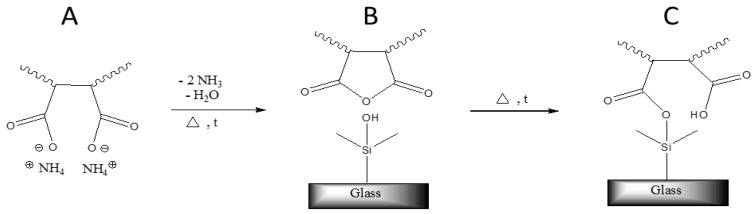
The chemical bounding of the terpolymer coated from aqueous ammonia solution with the glass surface.

Although an annealing step was not necessary to obtain large contact angles, it was of crucial importance to ensure a permanent adhesion of the polymers to the glass surface. Since it is known that anhydride groups can form silanol-esters with –Si–OH– groups present at the glass surface at elevated temperatures [14,15], it is assumed that polymers C1–C3 covalently link to the substrate by a similar mechanism (see Figure 1). 

Note that the contact angles of the dodecyl methacrylate (DMA) based copolymer coatings C3 (S1-C3 and S1-C3 *, Table 5) were also independent of annealing and only for 2–3 degrees lower than that of the samples C2. Both BMA and DMA copolymers contain the same fraction of fluorinated building blocks, hence their minor difference in surface properties is determined by the alkyl-methacrylate comonomer. The hydrophobicity is in principle equal (in the range of measurement error), but the higher oleophilicity of C3 seems to be a result of the presence of long alkyl chains as stated by the good solubility of C3 in hydrocarbons (see Table 4). 

*Emulsion of organic polymer solution in water.* A possible way to prepare waterborne systems from the investigated fluoropolymers is to prepare water based emulsions of polymer solutions in organic solvents [16]. Organic solvents can only be industrially applied if they are non-miscible with water, exhibit low volatility (i.e., high boiling points) and are non-hazardous with respect to human health and environment. However, for scientific purposes, other solvents like chloroform and HFX can also be employed. 

The formation of emulsions of C1, C2 and C3 was checked with chloroform (E1, E2, E4, E5, E15, E16), HFX (E3, E6), ester- (E7–E10), and hydrocarbon-solvents (E11–E14) according to Table 3. About 10 wt % of a copolymer was dissolved in an organic solvent that was emulsified either in pure water or in an SDS solution by means of an ultrasonic homogenizer. Emulsions obtained without SDS were found to be stable only for several days. Since detergent-free emulsions of chloroform in water break within seconds, this observation indicates that the terpolymers act as weak surfactants, which are, however, unable to stabilize oil droplets in water permanently. 

If *i*-butyl acetate was used as the auxiliary solvent (E7–E10, Table 3), the polymeric material precipitated within one week, most probably because of the slight solubility in water (6.7 g in 1000 g of water). Most probably, the solvent was taken up by the aqueous major phase, leaving back glassy/sticky polymer droplets that easily coagulate. An alternative explanation might be the partial hydrolysis of the maleic anhydride (MAH)moieties due to water, becoming dissolved in the organic phase.

This reaction could have created a polymer that was too hydrophilic to be soluble in the organic phase but still remained too hydrophobic to be soluble in water. This phenomenon was not observed with water immiscible auxiliary solvents. In case of more hydrophobic organic solvents like hexadecane, the dispersion is additionally stabilized by building up a counteracting osmotic pressure. Employment of a so-called “ultrahydrophobe”, a substance which is not able to migrate through the water phase is known to prevent Ostwald ripening [17]. This can be an explanation for the fact that emulsions E12 and E14 showed a liquid/liquid phase segregation, but no coalescence (see Table 3). 

Introducing 0.25 wt % SDS as the surfactant leads to stable emulsions with the exception of *i*-BuAc emulsions (E7, E9) where a partial precipitation of the polymer was still observed.

*Coating from emulsions.* In subsequent experiments, coatings have been prepared from water-emulsified chloroform-solutions of the P[R_F_-*co*-R_H_-*co*-MAH]. Emulsions E2 and E4 of the BMA based copolymers C1 and C2 in chloroform gave less hydrophobic or oleophobic surfaces than those obtained after spin-coating of polymers from chloroform solution (S1-C1 and S1-C2, Table 5). Comparative measurements of coatings obtained from emulsions E15 ([C3/CHCl_3_]/H_2_O) and E13 ([C3/C_16_H_34_]/H_2_O) revealed the latter to exhibit a larger contact angle against water (E15: Θ_H2O_ = 105°, E13: Θ_H2O_ = 108.5°). The angle was close to that of coating S1-C3 (Θ_H2O_ = 108°), obtained from a water-free chloroform solution. This comparison could not be made for BMA copolymers C1 and C2 due to their insolubility in hexadecane (see Table 4).

As was mentioned before, the organic solvent seems to plasticize the polymer during the deposition process, allowing the perfluoroalkyl-groups to move towards the air/film interface before the solvent evaporates [16]. When coating emulsions, the low-boiling solvent obviously evaporates first, leaving back a water rich polymer containing phase. Due to its incompatibility to water, the polymer partially vitrifies, hence the side chains become unable to orient properly and the measured contact angles are lower by 4–7 degrees (cf. S1-C1 vs. E2 and S1-C2 vs. E4 in Table 5). Note that this problem could not be solved by annealing.

Although the contact angles of hexadecane do not exceed 65°–67°, the oil did not adhere to the coatings. Upon tilting the glass plate, the droplets of hexadecane slid from the glass plates without leaving any oil trace on the surface. This is an important observation that shows the effectiveness of the coating as oil repellent. 

The above-mentioned explanation of the deposition mechanism is a hypothesis that needs to be further investigated. Such an investigation is not in the scope of the presented work.

Not only glass but also paper and anodised aluminium were covered with the emulsion and annealed at 120 °C for 12 h. Coatings prepared from E2 on aluminium substrates showed contact angles of water and hexadecane very close (±2°) to the values measured on coated glass (cf. Table 5, E2). On coating the emulsions E4, E7, E9, and E11 on aluminum, oil and water repelling surfaces were also obtained with Θ_H2O_ = 105°–110° and Θ_HD_ = 65°–70°.

The properties of the coating obtained on paper are different and the measured contact angles for both wetting liquids were lower (Θ_H2O_ = 100°–103° and Θ_HD_ = 59°–64° cf. Table 5) than in case of glass and aluminum. This phenomenon can be caused by penetrability of the paper. Probably, a large part of the copolymer penetrates the substrate together with the solvent, becomes absorbed on the cellulose fibers inside the structures of the paper and was not available to form a closed film. Tests to explore further the structure of the coatings (e.g., X-ray photoelectron spectroscopy (XPS) or scanning electron microscopy (SEM) were not performed.

*Preparation of aqueous solutions.* Since environmental regulations impede the use of Freon-113^®^ and other halogenated solvents in industrial applications and since organic solvents are no longer accepted for household users, aqueous formulations of surface modifying polymers are mandatory. As already shown by means of solubility tests, a spontaneous dissolution of the investigated copolymers in water and aqueous ammonia was not observed. In order to facilitate the process, isobutyl acetate (*i*-BuAc) was used as “auxiliary solvent” to swell the polymeric material and depress its glass transition temperature [18], prior to contact with aqueous ammonia solution. Hence, mixtures of terpolymers C1, C2 and C3 with *i*-BuAc (terpolymer:*i*-BuAc = 1:1, mass:mass) were suspended in 1% ammonia solution and stirred for at least 12 h. This treatment did not change the appearance of the copolymer. The application of ultrasound mechanically disrupted the gel-particles, but did not cause emulsification. The terpolymer could not be detected in the liquid phase, neither direct by evaporation of the filtrate, nor indirect by casting a drop of the liquid phase on a glass plate, followed by annealing and subsequent contact angle measurement. Any tested combination of heating, stirring as well as ultrasonication led in the best case to the dispersion of small particles with a strong tendency to agglomeration and sedimentation. 

The issue was settled by dissolving terpolymers in *i*-BuAc (0.5 g polymer in 10 g *i*-BuAc), followed by ultrasonication and subsequent standing of the mixture for seven days at room temperature. The spontaneously precipitated copolymer was separated by filtration and transferred to 1% aqueous ammonia. The mixture was homogenized by means of ultrasonication and stirred at elevated temperature for 30 min to yield a clear aqueous solution with polymer C2, however not with polymer C1 and C3. A picture of the clear solution of 2 wt % of C2 in aqueous ammonia (1%) is presented in Figure S1 A (Supplementary Information). Obviously, the composition of terpolymers C1 and C3 is not inside the “emulsification window”: it is assumed that the MAH-content of C1 (10 mol %) is too small to enable water solubility, while the long alkyl side chains of C3 impede the emulsion formation despite the large MAH content of 22%.

**Figure 2:**
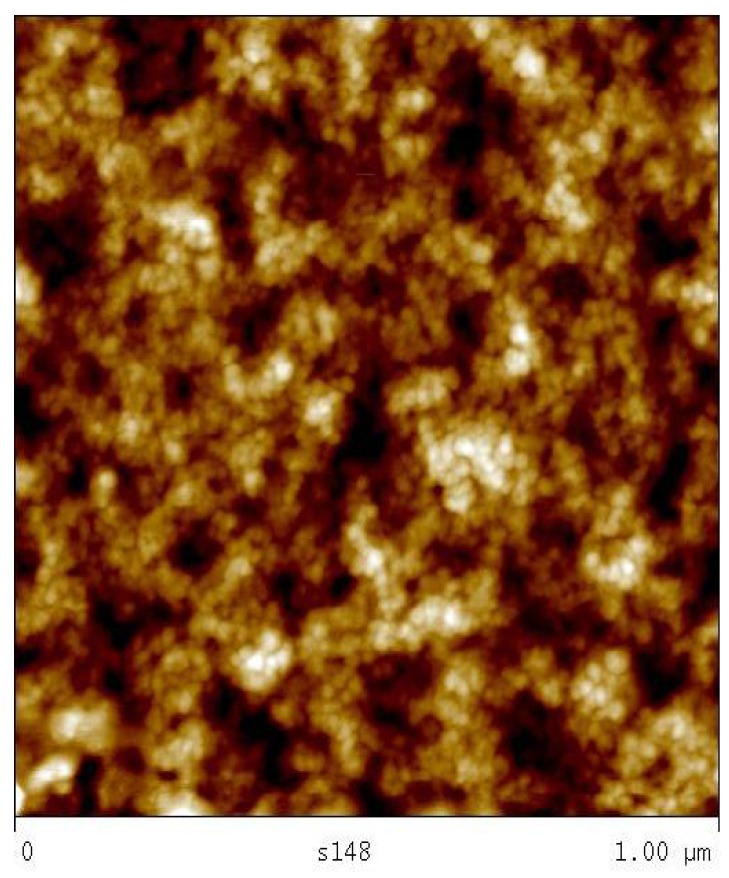
Solution (S 22) of polymer C2 2 wt % in aqueous ammonia (1%): AFM image.

The average diameter of the particles present in the aqueous dispersion as measured by means of a particle sizer is 50 nm and the particle size distribution is skewed towards larger diameters (see Figure S1C, Supplementary Information). A sample of the aqueous solution of C2 was further investigated by means of atomic force microscopy (AFM) after spin coating on mica (Figure 2). The size difference registered by means AFM and particle sizer methods can be explained by the accuracy of these methods and the size of measured particle population (entire in the case of particle sizer and partial for AFM). It is known that bimodal distributions obtained by AFM are registered as skewed monomodal distributions by a particle sizer [19], which might be valid in this case. The size of the spherical structures indicates that the particles are formed by more than one macromolecule. The number of the molecules in such particles was estimated assuming the density of the copolymer to be 1 g/cm^3^. A particle of 12 nm in diameter should hence consist of about seven polymer molecules, while a 20 nm particle should host 30 macromolecules. The structure of such a particle is schematically depicted as in Figure S1B (Supplementary Information). The pretreatment process described above generated associated structures and fluorinated and alkane side chains (thin black lines) are proposed to form a hydrophobic core that is shielded by the hydrophilic ammonium succinate groups (spheres) against the aqueous environment. The polymer backbone is depicted as a thick, black line.

**Scheme 3:**
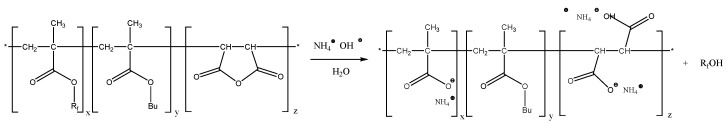
Hydrolysis of perfluorinated side chains in terpolymer (there is no direct evidence for this assumption!).

*Film formation and contact angles.* The aqueous “micellar solution” of the P[F8H2MA_0.2_-*co*-BMA_0.65_-*co*-MAH_0.15_] (C2) was used to prepare coatings. The contact angle against water and HD of such coating measured after annealing is Θ_H2O_ = 110° and Θ_HD_ = 71° (see S22-1, copolymer C2 in Table 6) and is in principle the same as in the case of the coating obtained from organic solution Θ_H2O_ = 110°, Θ_HD_ = 73° (see S1-1 for copolymer C2 in Table 6). The annealing at elevated temperature is especially important in the case of the coating obtained from aqueous ammonia solution where the anhydride ring was converted to carboxylic acid ammonium salt as depicted in Scheme 3.

It might be possible that the terpolymer became soluble because of partial hydrolysis of perfluorinated side chains during the long-term procedure of preparation of the aqueous solution. The decrease of the number of perfluorinated moieties and generation of additional hydrophilic carboxylic groups could enhance dissolution of the terpolymer in water. Scheme 3 depicts the hydrolysis of fluorinated methacrylate and salt formation of the remaining carboxylic acid as well as the salt formation upon hydrolysis of the succinic anhydride moiety.

Any long-chain perfluorinated alcohol possibly generated by hydrolysis could form a physisorbed hydrophobic layer on the surface of the coating causing oil- and water repellency. However, because of its physisorbed nature, this layer should be removable by rinsing the sample with a large excess of suitable solvent. Ethyl acetate was chosen as the most suitable because it is a good solvent for fluoro-alcohols but should not destroy the polymer coating. The washing after annealing gave unexpected results. The contact angle against water of the coating washed with ethyl acetate was 140° (see S22-2, Table 6) and was 30° higher than one measured for non-washed surface (S22-1). The contact angle measured against hexadecane did not change. This procedure was repeated for coating obtained from an organic solution for comparison purposes as no presence of free perfluorinated alcohol can be expected. The results obtained for coating prepared from solution were identical: the value against water for surface washed with ethyl acetate was 141° (see S1-2, C2), which was 31° higher than without washing (see S1-1, C2). The comparison between contact angle values for different coatings is summarized in Table 6.

**Figure 3:**
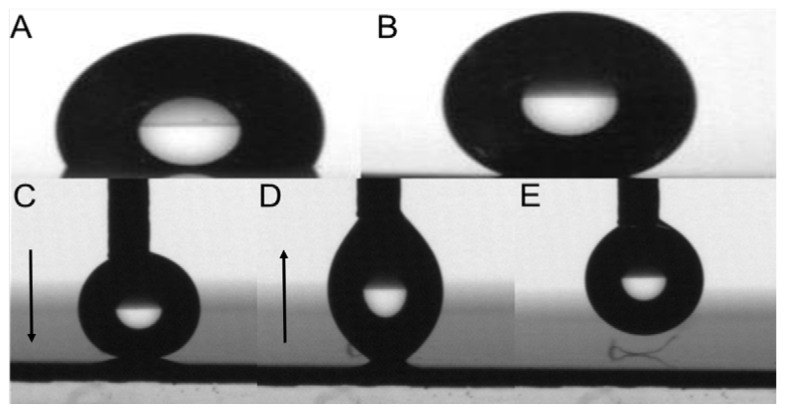
(**A**) water droplet on the glass coated with S 22 (Θ = 110°) and (**B**) on the glass coated with S 22 after rinsing with ethyl acetate (Θ = 140°) (both after annealing). The “ball jump motion” phenomenon—advancing (**C**) and receding (**D**,**E**) on anodised aluminium coated with S 22 after annealing and rinsing steps. The Arrows in Figure 3(**C**,**D**) indicate the motion of the cannula.

The remarkable difference between wettability by water of the coating made from water on the glass slide is very well depicted in Figure 3 before washing 110° (Figure 3A) and after washing with ethyl acetate 141° (Figure 3B).

Our hypothesis on the occurrence of this very high hydrophobicity of the surface that occurs despite previous hydrolysis can be described in the following manner: (I) the low molecular weight chain aligns perfectly at the surface; (II) heat-treatment causes chemisorption due to the ester formation with surface hydroxyl groups; and (III) the subsequent rinsing removes non-reacted perfluorinated alcohol leaving a perfect surface layer of covalently bound R_F_ chains.

To confirm or negate this possibility, the sample was rinsed with ethyl acetate prior to annealing. The contact angle of the coating obtained from aqueous dispersion and rinsed with ethyl acetate without annealing was measured against water, and it is significantly lower than this of annealed coating. The measured contact angle of 107° (see S22-3, Table 6) is close to the value obtained after annealing but without rinsing with ethyl acetate. This indicates that the assumed hydrolysis did not take place or is not significant. The obtained, lower value of the contact angle is most probably caused by insufficient reorganization of the terpolymer coated form aqueous dispersion at room temperature. Possibly, the behaviour after rinsing with suitable solvent is caused by removing layers of impurities from the coating by the solvent, as well as by giving the coating a second chance for orientation due to the liquids’ plastifying effect.

Measurement of contact angles of coating made from the aqueous solution of C2 on the anodized aluminium substrate faced unexpected problems. The water droplet that had to be settled on the coated substrate exhibits behavior similar to so-called “ball jump motion”. Because of the very low adhesion to the surface, the droplet receded together with the needle instead of settling on the substrate. Some adhesion of the droplet to the coating still can be observed; however, it is too weak to keep the droplet on the surface. The observed effect is therefore less pronounced than the “ball jump motion” reported by McCarthy et al. for super hydrophobic surfaces with contact angles >170° [20,21]. The above described observation is depicted in Figure 3C–E. 

Another possible explanation could be the roughness of the anodised aluminium. Unusual wettability involving very high contact angles of 150°–170° against water has been reported and explained as the influence of the surface roughness on the micro or nanometer scale [22,23,24]. 

It is important to remember that the measured contact angles in all cases are sessile drop measurements (in equilibrium with surface) and are neither advancing nor receding measurements and, due to this fact, the hysteresis has not been determined. Measurement of hysteresis is especially important in case the surface topology can play a significant role in the wettability of the coating [25]. This can be a subject of further investigation.

## 4. Conclusions

Fluorinated terpolymers of the composition P[R_F_MA-*co*-R_H_MA-*co*-MAH] (R_H_ = C_4_H_9_-, C_12_H_25_-, R_F_ = C_10_H_4_F_19_-) containing ca. 20 mol % of fluorinated monomer units can be dissolved in semi polar solvents like tetrahydrofuran, chloroform or ethyl acetate to give more than 20 wt % solutions. They exhibit good solubility in fluorinated solvents like HFX and Freon-113^®^. Upon incorporation of dodecyl-side chains (R_H_ = C_12_H_25_-), the polymer also becomes soluble in hydrocarbons. 

Coatings on glass obtained from polymer solutions show good water repellence (Θ = 108°–110°) and sufficient oil repellence (Θ = 67°–73°). The oleophobicity of the C3 terpolymer based on DMA is clearly lower (by 6°) than that based on BMA. Surprisingly annealing (a well-known method to enhance surface properties) has no effect on the wettability of thin films formed by the investigated copolymers. This is most probably caused by combination of the relatively low *T_g_* of the copolymers and the volatility of solvent enabling proper orientation of fluorinated chains to the surface.

Emulsification of organic solutions of the terpolymers resulted in unstable mixtures that demix within days, showing that self-emulsifying properties of investigated polymers (under testing conditions) are poor. Stable emulsions were obtained only in the presence of additional surfactant.

Water and hexadecane contact angles of coatings prepared from emulsions of organic solutions (CHCl_3_/H_2_O) are slightly worse, ranging from 103° to 105°, than those obtained from solutions (CHCl_3_) 108°–110°.

A stable aqueous solution was obtained only from C2 copolymer (P[F8H2MA_0.2_-*co*-BMA_0.65_-*co*-MAH_0.15_]). The amount of hydrophilic building blocks necessary for water solubility must be in balance with the perfluorinated building block and the right type of third alkyl monomer. 

Thin films coated from this solution yielded a water (Θ = 110°) and oil-repelling surface (Θ = 71°) and the water repellence was considerably enhanced by washing of the annealed coating with ethyl acetate (Θ = 140°) while the contact angle against hexadecane remained unchanged. This was observed also for coatings made from CHCl_3_ (141°). The observed effect is most probably caused by removing of an impurity layer.

The influence of the alkyl side chain on the orientation of fluorinated side chains (stratification) is not well pronounced and cannot be univocally concluded. However, the contact angles measured for C3 that contains dodecyl side chains are slightly lower for both wetting liquids. R_H_, R_F_, and MAH–Terpolymers of moderate fluorine content are versatile, flexible to handle materials that offer a wide range of applications for surface modification.

## Figures and Tables

**Table 1:** Characteristics of the investigated terpolymers.

Sample#	F8H2MA [mol %]	R_H_	R_H_-MA [mol %]	MAH [mol %]	*M*n [kg/mol]	*M*w/*M*n	*T*_g_ [°C]
C1	21	-C_4_H_9_	69	10	23.5	2.15	56
C2	20	-C_4_H_9_	65	15	36.1	1.80	58
C3	20	-C_12_H_25_	58	22	40.1	1.72	29

F8H2MA = 1*H*,1*H*,2*H*,2*H*-perfluorodecyl methacrylate, MAH = maleic anhydride, R_H_-MA = alkyl methacrylate, *M*_n_ = poly(methyl methacrylate) equivalent number average molar mass, *M*_w_/*M*_n_ = polydispersity, *T*_g_ = glass transition temperature.

**Table 2:** Solvents used for solubility and emulsifying investigations.

Solvent	Provider	Grade	Remarks
Chloroform	-	technical	distilled
THF	-	technical	distilled
Heptane	-	technical	distilled
Dodecane	Fluka	p.a.	-
Hexadecane	Fluka	p.a.	-
Butylacetate	Fluka	p.a.	-
MEK	Merck	for analysis	distilled
HFX	ABCR	-	-
Toluene	-	technical	distilled
Mesitylene	Merck	for synthesis	-
Cyclohexane	Merck	for synthesis	distilled
2-Octyl-1-dodecanol	Merck	for synthesis	-
Dipropylene glycolmonomethyl ether	Aldrich	97%	-
Freon-113^®^	Merck	for synthesis	-
White mineral oil	Aldrich	-	fl.p. > 110 °C
Mineral oil	Acros	-	fl.p. > 135 °C
Mineral oil for IR	Acros	-	fl.p. > 195 °C
Silicon oil	Fluka	-	fl.p. > 300 °C
NH_4_OH 25%	Merck	pure	-
Ethyl acetate	-	technical	distilled
Water	-	-	bi-distilled

THF = tetrahydrofuran, MEK = 2-butanone, HFX = 1,3-bis(trifluoromethyl) benzene.

**Table 3:** Emulsions of terpolymers in 20 mL water at room temperature after sonication for 10 min.

Sample#	Polymer	Solvent	SDS	Stability
	(200 mg)	(2 mL)	(50 mg)	
E 1	C1	chloroform	no	separation and coalescence within 2 days
E 2	C1	chloroform	yes	stable
E 3	C1	HFX	yes	stable
E 4	C2	chloroform	yes	stable
E 5	C2	chloroform	no	separation and coalescence within 2 days
E 6	C2	HFX	yes	stable
E 7	C2	*i*-butyl acetate	yes	precipitation of polymer within 2 weeks
E 8	C2	*i*-butyl acetate	no	precipitation of polymer within one week
E 9	C3	*i*-butyl acetate	yes	precipitation of polymer within one week
E 10	C3	*i*-butyl acetate	no	precipitation of polymer within one week
E 11	C3	*n*-heptane	yes	stable
E 12	C3	*n*-heptane	no	separation after 3 days but without coalescence
E 13	C3	*n*-hexadecane	yes	stable
E 14	C3	*n*-hexadecane	no	separation after 3 days but without coalescence
E 15	C3	chloroform	yes	stable
E 16	C3	chloroform	no	separation and coalescence within 2 days

HFX = 1,3-bis(trifluoromethyl) benzene.

**Table 4:** Solubility of the terpolymers in different solvents.

Sample#	Solvent	Solubility [wt %]		
		C1	C2	C3
S1	Chloroform	>20%	>20%	>20%
S2	THF	>20%	>20%	>20%
S3	*i*-Butylacetate	>20%	>20%	>20%
S4	MEK	>20%	>20%	>20%
S5	Ethyl acetate	>20%	>20%	>20%
S6	HFX	<14%	<13%	<12%
S7	Freon-113^®^	<10%	<10%	<8%
S8	Toluene	<1.8%	<1.9%	<19%
S9	Mesitylene	<1.7%	<1.7%	<20%
S10	Heptane	<0.01%	<0.01%	>20%
S11	Dodecane	<0.01%	<0.01%	<15%
S12	Hexadecane	<0.01%	<0.01%	<15%
S13	Cyclohexane	<0.01%	<0.01%	<12%
S14	White mineral oil	<0.01%	<0.01%	<0.01%
S15	Mineral oil	<0.01%	<0.01%	<0.01%
S16	Mineral oil for IR	<0.01%	<0.01%	<0.01%
S17	Silicon oil	<0.01%	<0.01%	<0.01%
S18	NH_4_OH 25%	<0.01%	<0.01%	<0.01%
S19	Water	<0.01%	<0.01%	<0.01%
S20	2-Octyl-1-dodecanol	swollen	swollen	swollen
S21	Dipropylene glycol	swollen	swollen	swollen
	monomethyl ether			
S22	1% aqueous ammonia	-	2% *	-

C1, C2: R_H_ = *n*-butyl, C3: R_H_ = dodecyl, THF = tetrahydrofuran, MEK = 2-butanone, HFX = 1,3-bis(trifluoromethyl) benzene, * only one concentration was tested.

**Table 5:** Contact angle Θ values measured on different substrates covered with C1, C2, and C3 copolymers coated from solutions and emulsions.

Sample#	Polymer	Solvent System	Glass	Θ_H2O_		Glass	Θ_HD_	
				Paper	Al		Paper	Al
S1-C1	C1	CHCl_3_	109° ± 2°	-	-	70° ± 1°	-	-
S1-C1 *	C1	CHCl_3_	108° ± 2°	-	-	71° ± 1°	-	-
S1-C2	C2	CHCl_3_	110° ± 2°	-	-	73° ± 1°	-	-
S1-C2 *	C2	CHCl_3_	110° ± 2°	-	-	73° ± 1°	-	-
S1-C3	C3	CHCl_3_	108° ± 2°	-	-	67° ± 1°	-	-
S1-C3 *	C3	CHCl_3_	108° ± 2°	-	-	67° ± 1°	-	-
S6-C1	C1	HFX	108° ± 2°	-	-	70° ± 1°	-	-
S6-C1 *	C1	HFX	108° ± 2°	-	-	70° ± 1°	-	-
E2	C1	CHCl_3_/H_2_O	105° ± 2°	-	-	67° ± 1°	-	-
E4	C2	CHCl_3_/H_2_O	103° ± 2°	-	-	70° ± 1°	-	-
E15	C3	CHCl_3_ in H_2_O	105° ± 2°	-	-	66° ± 1°	-	-
E13	C3	C_16_H_34_ in H_2_O	108.5° ± 1°	-	-	65° ± 2°	-	-
E2	C1	CHCl_3_/H_2_O	-	101° ± 1°	105° ± 1°	-	59° ± 1°	68° ± 1°
E4	C2	CHCl_3_/H_2_O	-	102° ± 1°	104° ± 1°	-	60° ± 1°	70° ± 1°
E7	C2	*i*-BuAc/H_2_O	-	100° ± 1°	110° ± 2°	-	62° ± 1°	69° ± 1°
E9	C3	*i*-BuAc/H_2_O	-	103° ± 1°	108.5° ± 1°	-	61° ± 2°	65° ± 2°
E11	C3	*n*-C_7_H_16_/H_2_O		102° ± 1°	108.5° ± 1°	-	64° ± 1°	65° ± 2°

C_16_H_34_ = hexadecane (HD), Al = anodised aluminium, *i*-BuAc = isobutyl acetate, * = no annealing.

**Table 6:** Surface properties of the coatings on glass, before and after washing with ethyl acetate.

Sample#	Solvent	Annealed	Rinsed EtAc	C1		C2		C3	
				Θ_H2O_	Θ_HD_	Θ_H2O_	Θ_HD_	Θ_H2O_	Θ_HD_
S1-1	CHCl_3_	Yes	No	109° ± 2°	70° ± 1°	110° ± 2°	73° ± 1°	108° ± 2°	67° ± 1°
S1-2	CHCl_3_	Yes	Yes	140° ± 4°	70° ± 1°	141° ± 4°	73° ± 2°	134° ± 4°	68° ± 3°
S22-1	Water	Yes	No	-	-	110° ± 2°	71° ± 1°	-	-
S22-2	Water	Yes	Yes	-	-	140° ± 5°	70° ± 1°	-	-
S22-3	Water	No	Yes	-	-	107° ± 2°	-	-	-

EtAc = ethyl acetate, HD = hexadecane.

## Supplementary Materials

The following are available online at www.mdpi.com/2073-4360/10/1/37/s1, Figure S1 (A) Solution (S 22) of polymer C2 2 wt % in aqueous ammonia (1%); (B) Proposed micellar structure of the fluorinated copolymer in water. Hydrophobic side chains form the core that is shielded by hydrophilic moieties; (C) Particle size (diameter) distribution of the aqueous dispersion of 2 wt % of fluorinated terpolymer C2.
