# Supplementary material for: Solubility, Emulsification and Surface Properties of Maleic Anhydride, Perfluorooctyl and Alkyl Meth-Acrylate Terpolymers
Source: Polymers (Basel). 2017 Dec 30;10(1):37. doi: 10.3390/polym10010037 (PMC6415136; doi:10.3390/polym10010037)
Supplement: Supplementary file 1 [file polymers-10-00037-s001.pdf]

# Solubility, Emulsification and Surface Properties of Maleic Anhydride, Perfluorooctyl and Alkyl Methacrylate Terpolymers.

**Marian Szkudlarek[1)], Uwe Beginn[2)*], Helmut Keul[1)], Martin Möller[1)*]**

[1] DWI Leibnitz Institute for Interactive Materials and Institute of Technical and Macromolecular Chemistry, RWTH Aachen University, Forckenbeckstraße 50, D-52056 Aachen, Germany, keul@dwi.rwth-aachen.de

[2] Universität Osnabrück, Institut für Chemie, OMC, Barbarastraße 7, D-49076 Osnabruck, Germany. E-mail: ubeginn@uni-osnabrueck.de

* Correspondence: ubeginn@uni-osnabrueck.de; Tel: +49-541-9692790; moeller@dwi.rwth-aachen.de; Tel: +49-241-8023302

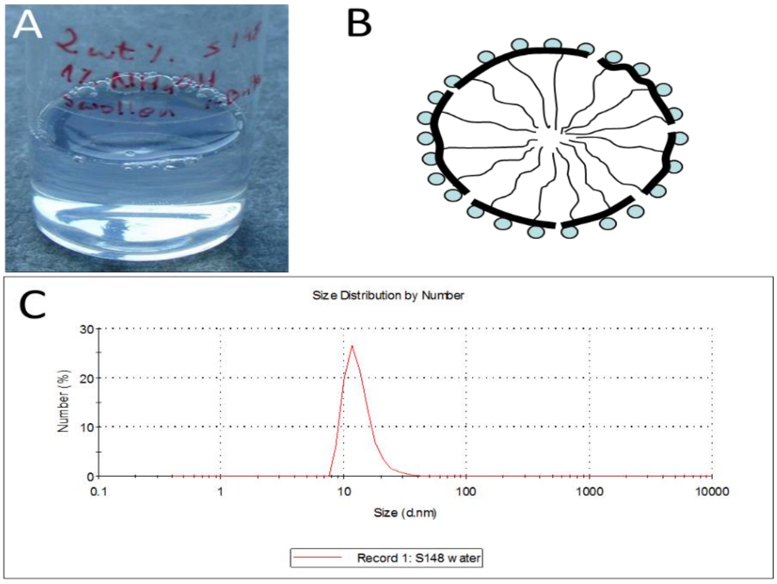


**Figure S1 A:** Solution (S 22) of polymer C2 2 wt% in aqueous ammonia (1%). **B:** Proposed micellar structure of the fluorinated copolymer in water. Hydrophobic side chains form the core that is shielded by hydrophilic moieties. **C:** Particle size (diameter) distribution of the aqueous dispersion of 2 wt% of fluorinated terpolymer C2 .
